# Monitoring small airway dysfunction in connective tissue disease-related interstitial lung disease: a retrospective and prospective study

**DOI:** 10.1186/s12890-023-02381-z

**Published:** 2023-03-20

**Authors:** Linrui Xu, Giacomo Sgalla, Faping Wang, Min Zhu, Liangyuan Li, Ping Li, Qibing Xie, Xiaoyan Lv, Jianqun Yu, Gang Wang, Huajing Wan, Luca Richeldi, Fengming Luo

**Affiliations:** 1 Department of Pulmonary and Critical Care Medicine, West China Hospital, Sichuan University, Chengdu, 610041 Sichuan P.R. China; 2 Laboratory of Pulmonary Immunology and Inflammation, Frontiers Science Center for Disease-Related Molecular Network, West China Hospital, Sichuan University, Chengdu, Sichuan P.R. China; 3 Clinical Research Center for Respiratory Disease, West China Hospital, Sichuan University, Chengdu, Sichuan P.R. China; 4 Division of Pulmonary Medicine, Fondazione Policlinico Universitario A. Gemelli IRCCS, Università Cattolica del Sacro Cuore, Rome, Italy; 5 Department of Rheumatology and Immunology, West China Hospital, Sichuan University, Chengdu, Sichuan P.R. China; 6 Department of Dermatology, West China Hospital, Sichuan University, Chengdu, Sichuan P.R. China; 7 Department of Radiology, West China Hospital, Sichuan University, Chengdu, Sichuan P.R. China

**Keywords:** Connective tissue disease associated interstitial lung disease, Pulmonary function, Small airway dysfunction, Treatment response

## Abstract

**Background:**

Small airway dysfunction (SAD), a hallmark of early lung function abnormality, is a major component of several chronic respiratory disorders. The role of SAD in patients with connective tissue disease-related interstitial lung disease (CTD-ILD) has not been explored.

**Methods:**

We conducted a two-parts (retrospective and prospective) study to collect pulmonary function tests from CTD-ILD patients. SAD was defined as at least two of the three measures (MMEF, FEF 50%, and FEF 75%) must be 65% of predicted values. Spearman correlation coefficient was used to evaluate association between SAD and other pulmonary function parameters. Mixed effects regression modeling analysis was used to assess response to treatment.

**Results:**

CTD-ILD patients with SAD and without SAD were compared in this study. In the retrospective study, pulmonary function tests (PFTs) from 491 CTD-ILD patients were evaluated, SAD were identified in 233 (47.5%). CTD-ILD patients with SAD were less smokers (17.6% vs. 27.9%, *p* = 0.007) and more females (74.3% vs. 64.0%, *p* = 0.015) than those without SAD. CTD-ILD patients with SAD had lower vital capacity (% predicted FVC, 70.4 ± 18.3 vs. 80.0 ± 20.9, *p* < 0.001) and lower diffusion capacity (% predicted DLCO, 58.8 ± 19.7 vs. 63.8 ± 22.1, *p* = 0.011) than those without SAD. Among 87 CTD-ILD patients prospectively enrolled, significant improvement in % predicted FVC was observed at 12-months follow-up (6.37 ± 1.53, *p* < 0.001 in patients with SAD; 5.13 ± 1.53, *p* = 0.002 in patients without SAD), but not in diffusion capacity and SAD parameters.

**Conclusion:**

In our cohort, about half of CTD-ILD patients have SAD, which is less frequent in smokers and more common in female patients. CTD-ILD patients with SAD have worse pulmonary function compared to those without SAD. Improvement of FVC but no improvement of SAD was observed in CTD-ILD patients after treatment.

**Supplementary Information:**

The online version contains supplementary material available at 10.1186/s12890-023-02381-z.

## Introduction

Interstitial lung disease (ILD) is the most common pulmonary manifestation of connective tissue diseases (CTDs), involving autoimmunity and multiple manifestations of respiratory complications affecting the airways, lung parenchyma, pleura, and respiratory muscles, and is associated with significantly increased morbidity and mortality of CTDs [1, 2]. The prevalence of ILD has been reported in 70–90% systemic sclerosis (SSc), 15–70% idiopathic inflammatory myopathies (IIM), 20–85% mixed connective tissue disease (MCTD), 4–68% rheumatoid arthritis (RA), 10–30% primary Sjögren’s syndrome (pSS), and 2–10% systemic lupus erythematosus (SLE) patients. Thus, early diagnosis and evidence-based management of ILD are crucial for better prognosis of CTD-ILD. Recently, several CTD-ILD guidelines or position statements from Japan, Korea, Australia and New Zealand were published, providing a framework to aid clinicians in ILD assessment and management [3–5]. Nevertheless, few prognostic determinants are available to assess the ILD progression in the context of CTD due to lack of understanding of the disease trajectory.

Among pulmonary function tests (PFTs), several key parameters have been used to monitor pulmonary disease progression and treatment response, including forced vital capacity (FVC) for lung volume, diffusion capacity for carbon monoxide (DLCO) for gas diffusion capacity, maximum mid expiratory flow (MMEF), 50% forced expiratory velocity (FEF50%) and 75% forced expiratory velocity (FEF75%) for small airway function [6–10]. Longitudinal measurement of PFTs is suggested for monitoring the progression of ILD in CTD-ILD patients in the newly published guidelines [3]. PFTs for CTD-ILD mainly reflect a “restrictive” ventilatory pattern and a reduced diffusion capacity. FVC and DLCO have been usually suggested as key parameters to assess the severity of ILD in CTD-ILD patients [2]. On the other hand, whether small airway function is worth to be assessed in CTD-ILD has remained unknown.

Small airways are defined as bronchial airways less than 2 mm in internal diameter, generally located from the eighth generation of airways to the respiratory bronchioles and account for 98.8% of the total lung volume (corresponding to approximately 4500 ml) [11]. Recent pathological studies demonstrated that bronchiolitis is identified in ILD, including idiopathic pulmonary fibrosis (IPF) [12–14], RA-ILD [5] and SS-ILD [5]. Thus, we hypothesized that SAD could be detected by PFTs in CTD-ILD patients and may be used as a prognostic parameter of pulmonary function in CTD-ILD patients. In the present study, we carried out retrospective and prospective studies of SAD in CTD-ILD, and provided evidence to highlight the needs for in-depth study of SAD in CTD-ILD patients.

## Methods

### Study design

The study was conducted at West China Hospital of Sichuan University (approval number 2019–246) and consisted of two parts. Part one was a cross-sectional study, in which the proportion of SAD in CTD-ILD and correlations between SAD indicators and lung function were retrospectively assessed. Part two was a cohort study, in which CTD patients who developed ILD were prospectively enrolled. Written informed consent was obtained from all participants in the prospective study and a waiver was obtained for the retrospective study.

### Subjects

In the retrospective cross-sectional study, participants were CTD-ILD patients admitted to the West China Hospital between January 2013 and December 2017 with a completion of PFTs. The demographic and clinical data, PFTs, and laboratory tests were extracted from hospital electronic medical records. The diagnosis of CTD-ILD was established according to current international guidelines [15–20]. Patients were excluded from the study for the following reasons: (1) age below 18 years; (2) pulmonary function tests not performed or not available; (3) acute exacerbation of CTD-ILD one month ago before collected PFTs; (4) concomitant pulmonary diseases, such as infection, pulmonary embolism, pneumothorax, chronic obstructive pulmonary disease and ILD caused by pneumoconiosis, inhalation of organic matter and other causes; (5) other concomitant diseases that could interfere with respiratory function and/or quality of life, such as severe cerebrovascular disease, severe heart failure caused by cardiovascular disease, severe renal disease. The interstitial lung disease–gender-age-physiology (ILD-GAP) index was evaluated and collected for all participants [21]. Two respiratory physicians who had finished standardized training were responsible for the data collection and evaluation.

In the prospective cohort study, patients diagnosed with CTD-ILD were consecutively recruited between March 2019 and March 2022. Multi-disciplinary follow-up evaluation was routinely performed for each patient about every 6 ± 2 months to manage treatment, according to expert consensus and clinical guidelines [5]. At each visit, symptoms and quality of life were assessed using standardized questionaries: Borg scale and modified Medical Research Council (mMRC Scale) for dyspnea, Visual Analog Scale (VAS) and Leicester Cough Questionnaire (LCQ) for cough, and Short Form of Quality-of-Life scale (SF-36). Patients were excluded according to the exclusion criteria defined in the retrospective study.

### PFTs and SAD evaluation

PFTs was performed by two trained technicians with over 20 years of experience using a full MasterScreen PFT System (Jaeger Corp. Germany) and accordingly to the American Thoracic Society (ATS)/European Respiratory Society (ERS) guidelines [22]. FEV_1_, FVC, FEV_1_/FVC, MMEF, FEF50, FEF75 and DLCO were measured as percentages of predicted values. Diffusion capacity was considered abnormally low under 80% of predicted [23]. The prediction equations and reference values were based on a large population study of normal spirometry values in a Chinese population aged 4–80 years [24], and difference of these values compared to the ones for Caucasian population are presented in Table S1. PFTs values were used to evaluate the presence of small airway dysfunction (SAD), based on clinical guidelines and a Chinese nation-wide population research on SAD [8]. MMEF, FEF 50%, and FEF 75% were calculated from PFTs measurements. SAD was defined according to the current Chinese population study [8, 25, 26]: at least two of the three measures (MMEF, FEF 50%, and FEF 75%) must be 65% of predicted values.

### Statistical analysis

Statistical analyses were performed using SPSS (version 21.0, IBM SPSS, Chicago, IL). To compare differences between groups, we used Chi-squared or Fisher exact test for categorical variables, and Student’s t or Wilcoxon’s signed-rank test for continuous variables. To identify risk factors associated with the presence of SAD, univariate and multivariate logistic regressions were performed. Correlations were calculated using Spearman correlation coefficient. Mixed effects regression modeling analysis of variance was performed to evaluate changes over time. Mixed ANOVA was performed to evaluate interaction between SAD and trends of pulmonary function. All *p* values were two-sided and significance was set to 0.05.

## Results

### Demographic and clinical characteristics

**Fig. 1:**
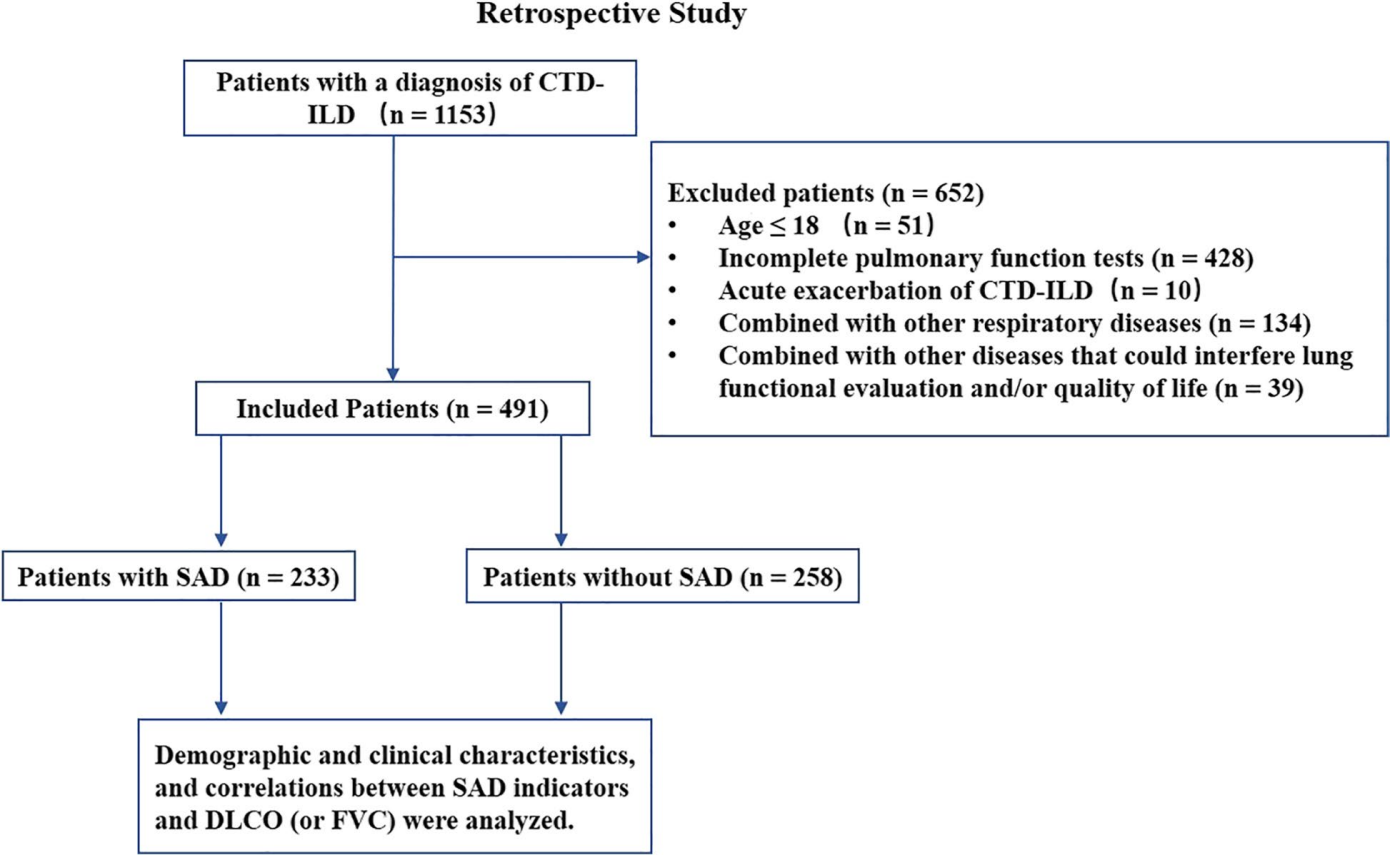
Flowchart of the retrospective study

**Fig. 2:**
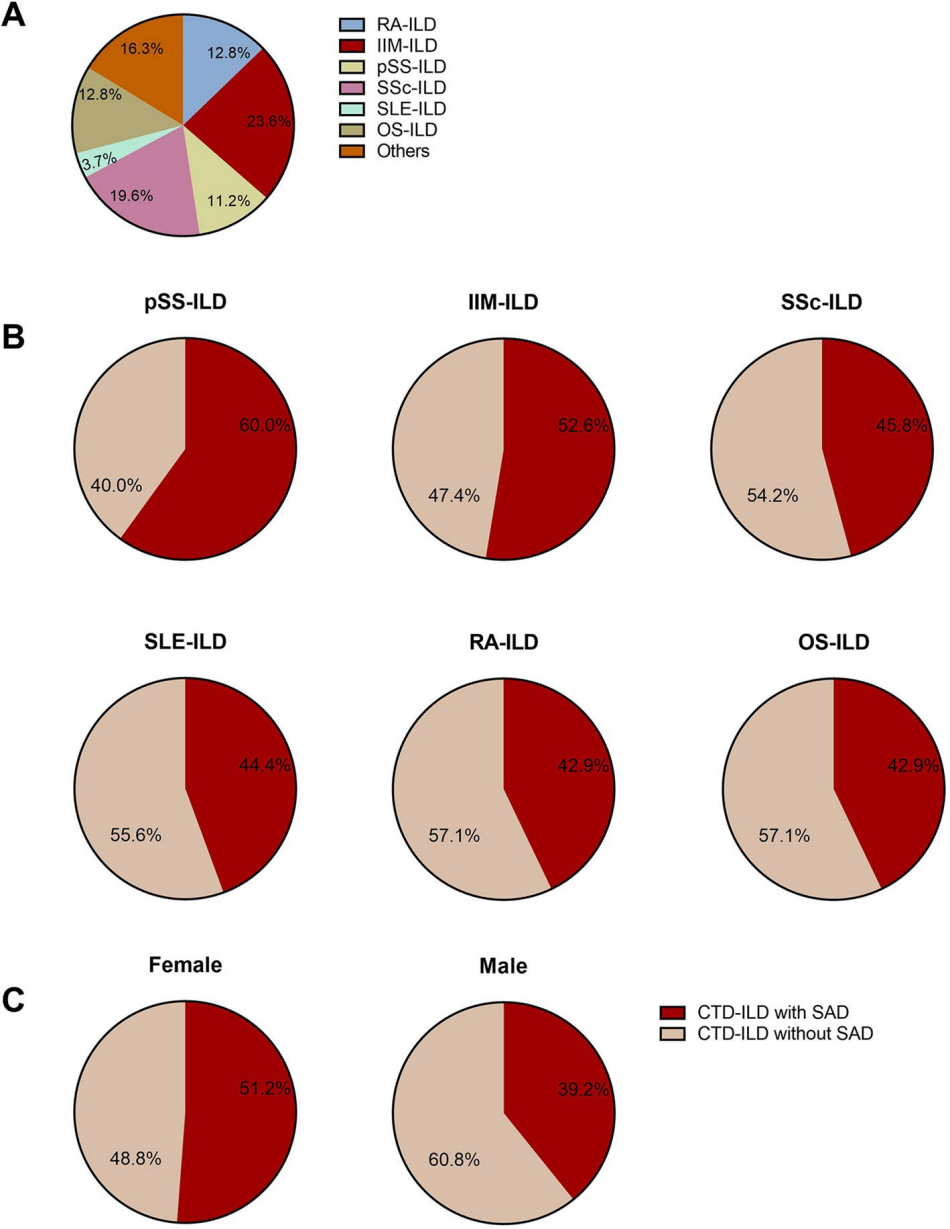
Types of CTD-ILD for retrospective data and proportion of SAD in different groups. **A** All the types of CTD-ILD for retrospective study that included in our research, including 116 IIM-ILD (23.6%), 96 SSc-ILD (19.6%), 63 RA-ILD (12.8%), 55 pSS-ILD (11.2%), 18 SLE-ILD (3.7%), 63 OS-ILD (12.8%) and 80 other types of CTD (16.3%). **B** Proportion of SAD in different types of CTD-ILD. SAD was found in 60.0% pSS-ILD, 52.6% IIM-ILD, 45.8% SSc-ILD, 44.4% SLE-ILD, 42.9% RA-ILD, and 42.9% OS-ILD. **C** Proportion of SAD in different gender. SAD was found in 51.2% female patients and 39.2% male patients

In the part one retrospective study, 491 patients with CTD-ILD were included (Fig. 1 and Table S2): 116 patients had idiopathic inflammatory myopathies-related interstitial lung disease (IIM-ILD), 96 systemic sclerosis-related interstitial lung disease (SSc-ILD), 63 rheumatoid arthritis-related interstitial lung disease (RA-ILD), 55 primary Sjogren’s syndrome-related interstitial lung disease (pSS-ILD), 18 systemic lupus erythematosus-related interstitial lung disease (SLE-ILD), 63 overlap syndrome-related interstitial lung disease (OS-ILD) and 80 other types of CTD (Table S2, Fig. 2A). Fibrotic pattern was detected in all patients: 231 nonspecific interstitial pneumonia (NSIP), 73 usual interstitial pneumonia (UIP), 9 organizing pneumonia (OP), 4 lymphocytic interstitial pneumonia (LIP) and 174 other patterns of CTD-ILD. As for ILD-GAP index, 405 patients had 0–1 score, 78 had 2–3 scores, and 8 had 4–5 scores. Mean age was 54.4 ± 13.0 years, 68.8% were female, 23.0% had a smoking history.

PFTs of 491 patients were assessed. Most (79.8%) patients were identified to had impaired diffusion capacity and 233 (47.5%) had SAD. The highest proportion of SAD was among patients with pSS-ILD (Fig. 2B): in our population, patients with SAD had a lower prevalence of current and former smokers (17.6% vs. 27.9% in patients without SAD, *p* = 0.007) and heavy smoking history (smoking ≥ 20 pack-years, 11.6% vs. 18.2% in patients without SAD, *p* = 0.039), while BMI values were similar between the two groups. However, no difference was found in the patterns and ILD-GAP index of the CTD-ILD between the two groups (Table 1). Patients with SAD were more likely to be female (74.2% vs. 64.0% in patients without SAD, *p* = 0.015) (Table 1). The prevalence of SAD in female patients was significantly higher (51.2% vs. 39.2% in males, *p* = 0.020) (Fig. 2C).

**Table 1:** Comparison of demographic characteristics among CTD-ILD patients with and without SAD

	SAD (*n* = 233)	No SAD (*n* = 258)	p
Age, (y)	53.4 ± 12.8	55.3 ± 13.2	0.103
Female, n (%)	173(74.2%)	165(64.0%)	0.015
BMI, (kg/m2)	23.0 ± 3.5	22.2 ± 3.5	0.095
Ever smoker, n (%)	41(17.6%)	72(27.9%)	0.007
Former smoker, n (%)	21(9.0%)	54(20.9%)	< 0.001
Current smoker, n (%)	20(8.6%)	18(7.0%)	0.506
≥ 20 pack-years, n (%)	27(11.6%)	47(18.2%)	0.039
ILD duration, (y)	1.6 ± 2.3	1.4 ± 2.2	0.293
CTD duration, (y)	4.3 ± 6.4	4.1 ± 6.5	0.786
Patterns of CTD-ILD			
NSIP	114 (49.7%)	117 (45.3%)	0.324
UIP	31 (13.3%)	42 (16.3%)	
OP	7 (3.0%)	2 (0.8%)	
LIP	2 (0.8%)	2 (0.8%)	
Others	79 (33.9%)	95 (36.8%)	
ILD-GAP index			
0–1	188 (80.7%)	217 (84.1%)	
2–3	41 (17.7%)	37 (14.3%)	0.577
4–5	4 (1.6%)	4 (1.6%)	

Data are presented as n (%) or means ± SD. *SD* Standard deviation, *BMI* Body mass index

At univariate regression analysis, gender and smoking status were significantly associated with SAD, as shown in Table S3. Although BMI was not associated with SAD in our cohort, increased BMI is known as a risk factor for SAD [8]. At multivariate analysis including age, sex, smoking status and BMI, female sex (OR = 2.170, 95% CI 1.204–3.914, *p* = 0.01) and older age (OR 1.025, 95% CI 1.005–1.046, *p* = 0.017) were found to be independently associated with SAD (Table S3).

### Association of SAD and pulmonary-function

**Fig. 3:**
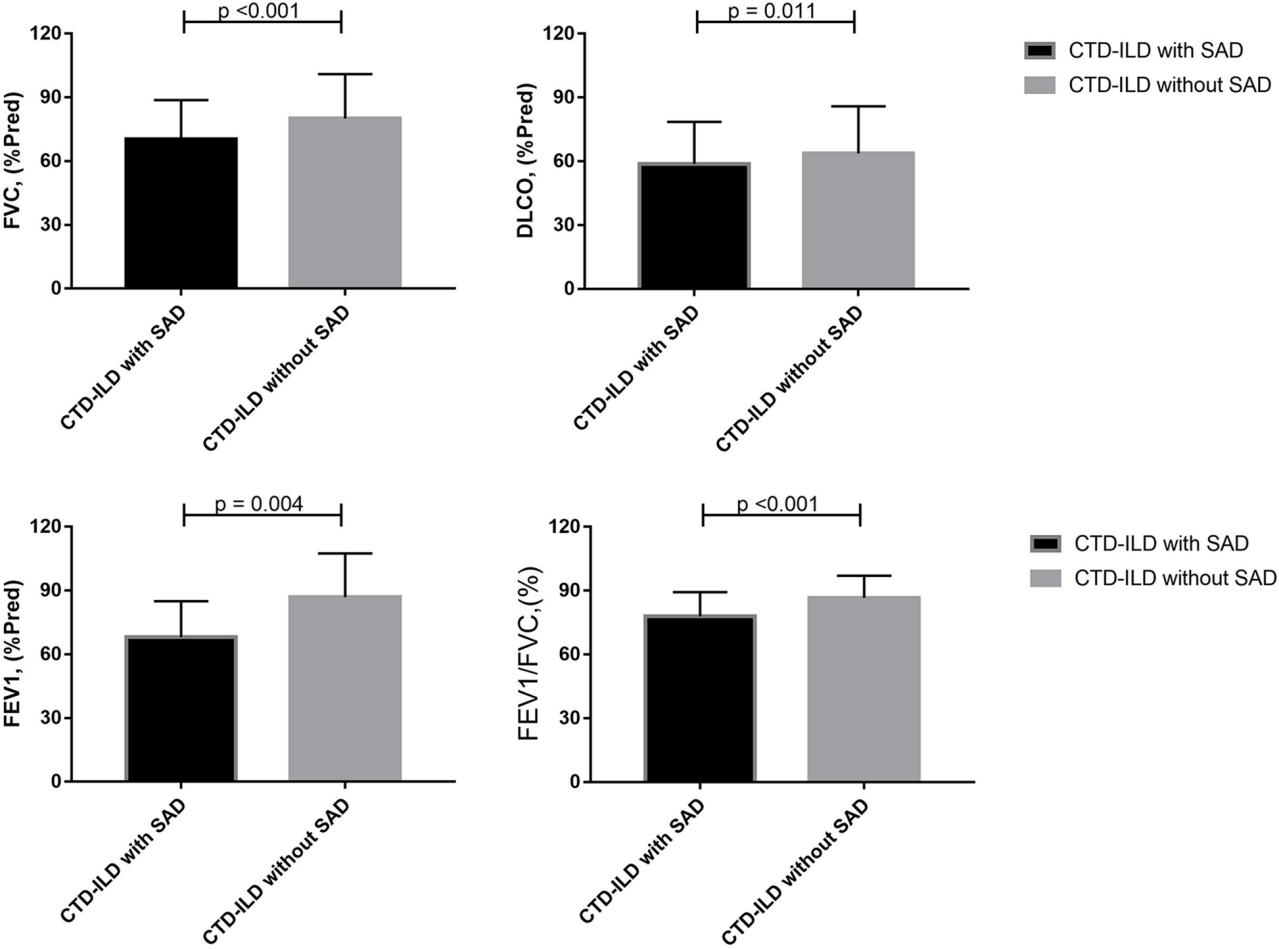
Comparison of pulmonary function tests between CTD-ILD patients with and without SAD for retrospective data. CTD-ILD patients with SAD (*n* = 233) showed worse pulmonary function values, compared to those without SAD (*n* = 258): FVC% (70.4 ± 18.3 vs. 80.0 ± 20 respectively, *p* < 0.001), DLCO% (58.8 ± 19.7 vs. 63.8 ± 22.1 respectively, *p* = 0.011), FEV1% (68.1 ± 16.9 vs. 86.9 ± 20.5 respectively, *p* = 0.004), FEV1/FVC% (78.0 ± 11.4 vs. 86.7 ± 10.4 respectively, *p* < 0.001)

Overall, CTD-ILD patients with SAD (*n* = 233) showed worse pulmonary function values, compared to those without SAD (*n* = 258), including lower % predicted FVC (70.4 ± 18.3 vs. 80.0 ± 20 respectively, *p* < 0.001). DLCO (58.8 ± 19.7 vs. 63.8 ± 22.1 respectively, *p* = 0.011), FEV1 (68.1 ± 16.9 vs. 86.9 ± 20.5 respectively, *p* = 0.004), FEV1/FVC (78.0 ± 11.4 vs. 86.7 ± 10.4 respectively, *p* < 0.001) (Fig. 3). A statistically significant correlation was found between SAD indicators (MMEF%, FEF75%, and FEF50%) and FEV_1_/FVC% (*r* = 0.571, *p* < 0.001, *r* = 0.564, *p* < 0.001 and r = 0.523, *p* < 0.001, respectively), as well as with FVC% (*r* = 0.247, *p* < 0.001, *r* = 0.202, *p* < 0.001 and *r* = 0.257, *p* < 0.001, respectively) and FEV1% (*r* = 0.516, *p* < 0.001, *r* = 0.478, *p* < 0.001 and *r* = 0.507, *p* < 0.001, respectively). Two of the functional parameters used to define SAD (MMEF% and FEF50%) showed significant, although weak correlation with DLCO% values (*r* = 0.108, *p* = 0.019 and *r* = 0.097, *p* = 0.035, respectively) (Table 2).

**Table 2:** Correlations between the indicators of SAD and other pulmonary function parameters

	**MMEF%**	**FEF75%**	**FEF50%**
**FVC%**			
rho	0.247	0.202	0.257
*p*-value	< 0.001	< 0.001	< 0.001
**FEV1/FVC%**			
r ho	0.571	0.564	0.523
*P*-value	< 0.001	< 0.001	< 0.001
**FEV1%**			
rho	0.516	0.478	0.507
*p*-value	< 0.001	< 0.001	< 0.001
**DLCO%**			
rho	0.108	0.077	0.097
*p*-value	0.019	0.095	0.035

*FEV1* Forced expiratory volume in the first second, *FVC* Forced vital capacity, *DLCO* Diffusion capacity for carbon monoxide, *MMEF* Maximum mid-expiratory flow, *FEF* Forced expiratory flow

### Association of SAD and scales

**Fig. 4:**
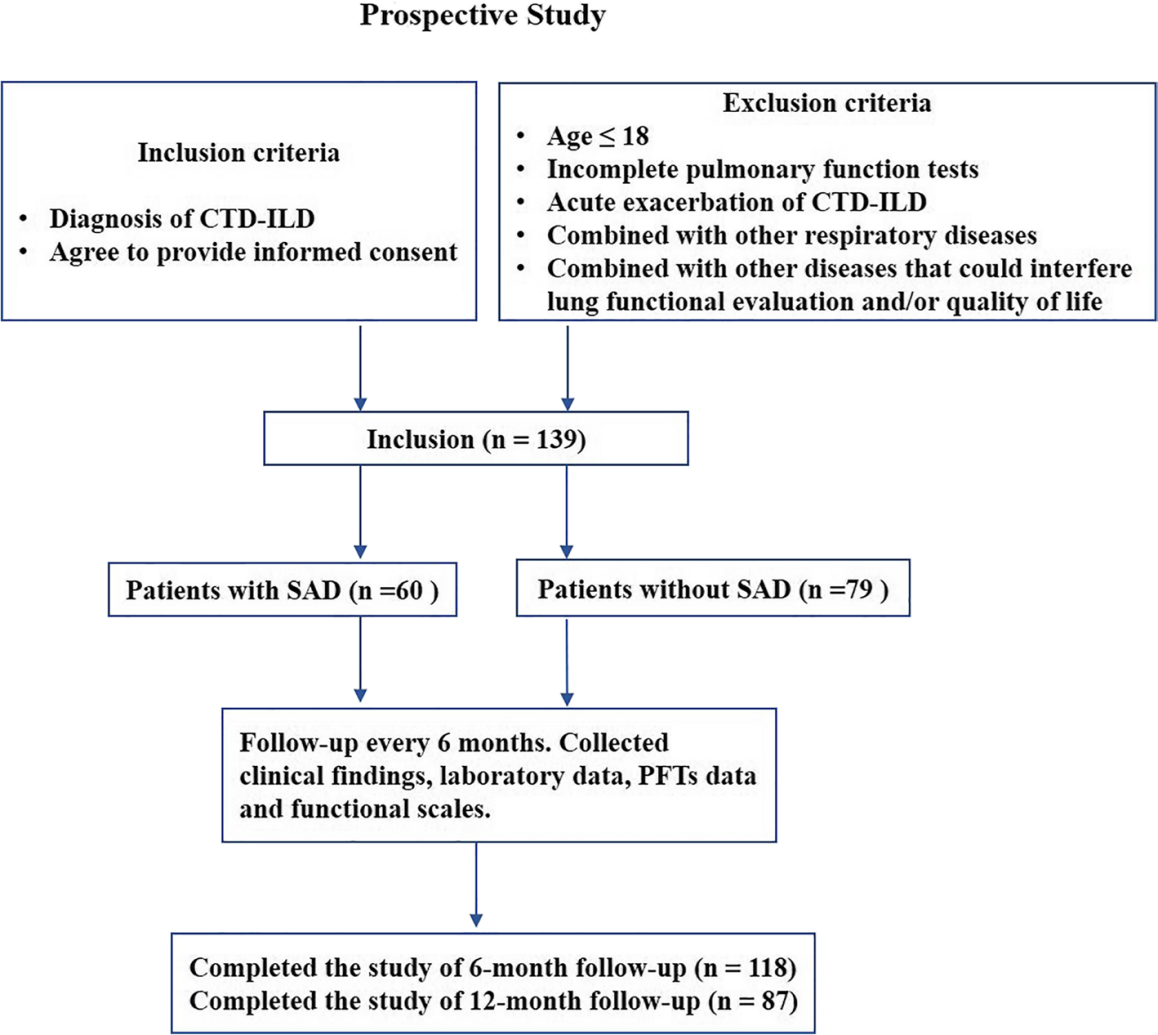
Flowchart of prospective study

In the part two prospective study (Fig. 4), 139 newly diagnosed CTD-ILD patients were enrolled. Demographic and clinical characteristics of these patients are shown in Table S4. Confirming the findings from the retrospective cohort, patients with SAD were more likely to be female (93.3% vs. 75.9% compared to patients without SAD, *p* = 0.006) and less likely to be current and former smoker (6.7% vs. 22.8% compared to patients without SAD, *p* = 0.010). All 139 patients received multi-disciplinary care, treatment was provided according to expert consensus and clinical guidelines [5], and follow-up evaluation was routinely performed every 6 months.

All the parameters of SAD (MMEF%, FEF75% and FEF50%) were negatively associated with mMRC score (*r* = -0.249, *p* = 0.003, *r* = -0.230, *p* = 0.007 and *r* = -0.273, *p* = 0.001, respectively) and positively associated with general health of Short Form of Quality-of-Life scale (*r* = 0.249, *p* = 0.003, *r* = -0.206, *p* = 0.015 and *r* = -0.243, *p* = 0.003, respectively). Two of parameters of SAD (MMEF% and FEF50%) showed significant correlation with Borg score (*r* = -0.172, *p* = 0.043 and *r* = -0.241, *p* = 0.004, respectively) (Table 3). At the same time, other PFT values (FVC% and DLCO%) were also negatively associated with mMRC score (*r* = -0.364, *p* = 0.001 and *r* = -0.368, *p* < 0.001), Borg score (*r* = -0.344, *p* = 0.001 and *r* = -0.380, *p* < 0.001). FVC% was showed significant correlation with VAS score and LCQ score (Table S5).

**Table 3:** Correlations between the indicators of SAD and symptom scale scores

	**MMEF%**	**FEF75%**	**FEF50%**
**mMRC**			
rho	-0.249	-0.230	-0.273
*p*-value	0.003	0.007	0.001
**Borg**			
rho	-0.172	-0.139	-0.241
*P*-value	0.043	0.103	0.004
**VAS**			
rho	-0.015	0.013	-0.031
*p*-value	0.862	0.881	0.713
**LCQ**			
rho	0.052	0.052	0.062
*p*-value	0.575	0.573	0.499
**GH**			
rho	0.249	0.206	0.247
*p*-value	0.003	0.015	0.003

*MMEF* Maximum mid-expiratory flow, *FEF* Forced expiratory flow, *mMRC* Modified medical research council for dyspnea, *VAS* Visual analog scale, *LCQ* Leicester cough questionnaire for cough, *GH* General health of short form of quality-of-life scale

### Treatment response

**Fig. 5:**
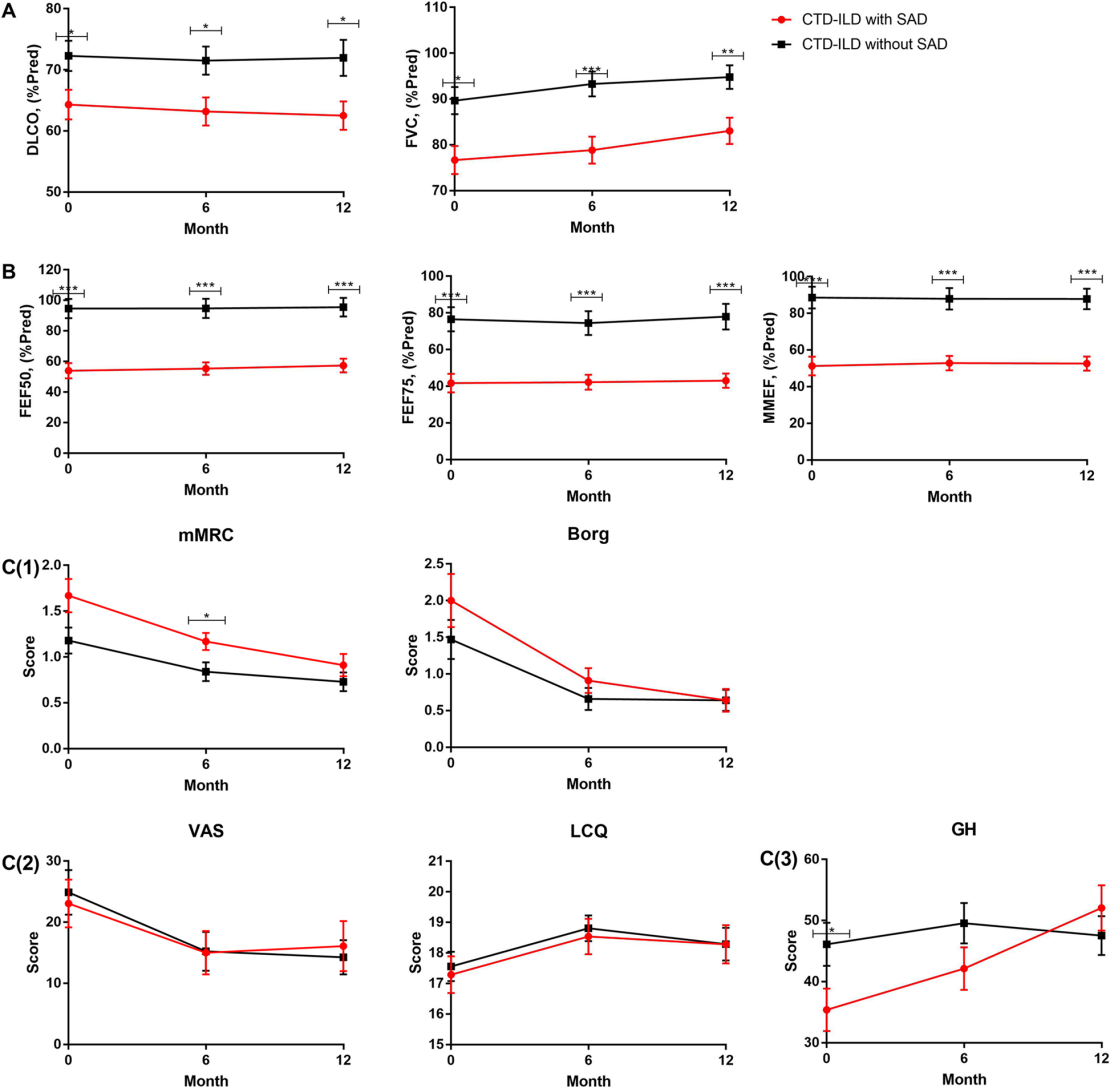
Changes of lung function and symptom scale scores during 12-months follow-up. **A** DLCO% and FVC%; (**B**) SAD indicators;(C(1)) Dyspnea symptom based on mMRC and Borg score; (**C**(2)) Cough symptom based on LCQ and VAS score; (**C**(3)) Overall health score based on general health of SF-36 Quality-of-Life score. Figure legend: FVC: forced vital capacity; DLCO diffusion capacity for carbon monoxide; MMEF, maximum mid-expiratory flow; FEF, forced expiratory flow; mMRC, modified Medical Research Council for dyspnea; VAS, Visual Analog Scale; LCQ, Leicester Cough Questionnaire for cough; GH, general health of Short Form of Quality-of-Life scale

Among the 139 CTD-ILD patients enrolled in the prospective cohort, average number of follow-up pulmonary function tests per patient was 2.47. 87 (62.6%) completed the 12-month follow-up and were included in the longitudinal analysis. Among the 87 CTD-ILD patients, 36 patients (41.7%) had SAD. Treatment data (treatment including immunosuppressive therapy, antifibrotic therapy, and/or antioxidant therapy) for CTD-ILD with and without SAD were collected (Table S6), and no significant imbalances regarding therapy were found between the two groups. Mixed effects regression modeling analysis showed that CTD-ILD patients with SAD had significantly increased FVC% at 12-months (change in % predicted FVC 6.37 ± 1.52, 95% CI 2.883–9.856, *p* < 0.001). CTD-ILD patients without SAD had significant increased FVC% versus baseline at both 6-months (change in % predicted FVC 3.62 ± 1.53, 95% CI 0.152–7.095, *p* = 0.039) and 12-months ((change in % predicted FVC 5.13 ± 1.53, 95% CI 1.656–8.599, *p* = 0.002). No improvement of diffusion function (DLCO%) and small airway dysfunction (FEF50, FEF75 and MMEF) were identified in either CTD-ILD patients with or without SAD (Fig. 5(A) (B)) (Table S7). Mixed ANOVA evaluating group differences in % predicted FVC across time showed that there was no interaction between SAD and trends of pulmonary function (F = 0.743, *p* = 0.447), indicating that the presence of SAD did not affect changes of FVC. To evaluate the treatment response among different CTD-ILDs, we compared with SAD and without SAD in different CTD-ILD subgroups using Mixed ANOVA, including IIM-ILD (FVC%, F = 0.984, *p* = 0.357; DLCO%, F = 0.712, *p* = 0.457), pSS-ILD (FVC%, F = 0.054, *p* = 0.850; DLCO%, F = 1.998, *p* = 0.229), SSc-ILD (FVC%, F = 0.944, *p* = 0.944; DLCO%, F = 2.263, *p* = 0.151) and OS-ILS (FVC%, F = 0.316, *p* = 0.615; DLCO%, F = 0.225, *p* = 0.713). All the subtype analysis revealed the presence of SAD did not affect changes of FVC or DLCO (Table S8). As for quality of life, improvement of symptoms was observed in all the CTD-ILD patients at 12 month follow up time.

CTD-ILD patients with SAD had lower FVC values at all the three points: baseline (76.7 ± 18.5 vs. 89.7 ± 21.3 in patients without SAD, p = 0.001), 6-month follow-up (78.9 ± 17.6 vs. 93.3 ± 19.2 in patients without SAD, *p* = 0.001) and 12-month follow-up (83.1 ± 17.2 vs. 94.8 ± 18.5 in patients without SAD, *p* = 0.001). (Fig. 5A). CTD-ILD patients with SAD also had lower diffusion capacity values at all the three points, baseline (64.3 ± 14.6 vs. 72.3 ± 17.8 in patients without SAD, *p* = 0.017), 6 (63.2 ± 13.8 vs. 71.5 ± 16.5 in patients without SAD, *p* = 0.017) and 12 months follow-up (62.5 ± 13.9 vs. 72.0 ± 21.2 in patients without SAD, *p* = 0.017). Besides, CTD-ILD patients with SAD showed more severe dyspnea based on the mMRC score (1.28 ± 0.818 vs. 0.78 ± 0.786, *p* < 0.001, *N* = 118) at 6-month (Fig. 5). No difference in dyspnea (according to mMRC and Borg score), and cough (according to VAS and LCQ score) were identified at any time point across 12 months between CTD-ILD patients with SAD and those without (Fig. 5C (1)(2)(3)).

## Discussion

This study represents to our knowledge the largest assessment of SAD in CTD-ILD patients using PFTs. We showed that SAD is present in almost half of patients with CTD-ILD, and is associated with worse lung function in CTD-ILD patients. On the other hand, the presence of SAD did not seem to affect response to treatment in these patients.

SAD is considered a functional hallmark of airway diseases, including COPD and asthma [27, 28], which is identified by PFTs in at least 75% of COPD patients [28] and 50–60% of adult asthma patients [27]. Studies demonstrated that longitudinal evaluation of SAD by PFTs plays indispensable roles in asthma/COPD control, including assessing disease severity, monitoring disease progression and evaluating treatment response [26, 29]. Recent studies suggest small airways abnormalities is an early manifestation of ILD [12, 13], and that small airway damage could affect lung function [30]. Zhang et al. have found that 30% of IPF patients had comorbid SAD, and the mortality risk of these patients was significantly higher compared with the non-SAD group [31]. Small airways disease could occur in CTDs like RA and pSS, which may be isolated or associated with ILD [32]. Nevertheless, there is very limited evidence to support whether evaluation of SAD by PFTs is worth for CTD-ILD monitoring and treatment evaluation. In the present study, we assessed the SAD in 491 CTD-ILD patients who were admitted to West China Hospital between January 2013 to December 2017, and detected the presence of SAD in almost half (47.5%) by PFTs, indicating that SAD is a frequent finding in CTD-ILD. This concept is also supported by recent pathological studies demonstrating that small airway abnormalities, including bronchitis/bronchiolitis, were present in CTD-ILD [5]. Consistently with previous studies showing that pSS are more likely to have small airways disease involvement compared with other subtypes of CTDs [33, 34], the highest SAD proportion (60%) was detected in pSS-ILD.

In previous studies, smoking and increased BMI were identified to be risk factors of SAD in healthy adults or COPD/ asthma patients [8, 35]. Interestingly, in our cohorts SAD was less common in smokers. Previous studies demonstrated that cigarettes impacts both innate and adaptive immunity by attenuation of defensive immunity [36]. Besides, nicotine in cigarettes may be anti-inflammatory. Infusions of nicotine could decreased immune cell influx and reduced pro-inflammatory cytokine release [37]. Smoking alters the distribution of T-helper cells, particularly shifting the balance between Th1, Th2 and Th17 cells [38], and reduces the proliferation of cytotoxic T lymphocytes [39], thereby influencing cytokine release. A recent cohort study has suggested smoking was associated with a reduced risk of severe COVID-19 [40], which may be influenced by the mechanism above. Whether smoking participates in inhibiting the overactive immune response and inflammatory cytokine release in CTD-ILDs is worth to be further explored. CTD is reported to be a female predominant disease [41]. In agreement with this, we found that CTD-ILD patients with SAD had a higher female rate and showed that female gender is an independent risk factor of SAD in CTD-ILD.

Previous studies implicate that small airway abnormalities may be an indicator of poorer prognosis of ILD, especially in IPF. For example, studies by Goto et al. demonstrated that FEF75/ FEF75 ratio, the PFTs parameter implicating SAD, may be used as one of the predictors of mortality [42]. Zhang et al. found IPF patients with SAD are related with higher mortality risk than those without SAD by using the same SAD definition as our study method [31]. Studies by Ikezoe et al. and Stijn et al. identified that small airway loss occurs in IPF and is associated with fibroblastic foci [12, 13]. In the present study, we found that CTD-ILD with SAD was associated with lower vital capacity and lower diffusion capacity in both retrospective and prospective cohorts. In addition, we carried out the first longitudinal assessment of SAD in CTD-ILD patients who were recruited for multidisciplinary care based on current clinical guidelines. We found that pulmonary function and symptoms improved over time after treatment in both CTD-ILD patients with SAD and without SAD, but not DLCO and small airway function parameters (MMEF5%, FEF50%, FEF75%). Notably, disease trajectory in these patients was not affected by the presence of SAD, which did not impact the functional improvement during the follow up. As such, our findings suggest that while SAD in CTD-ILD correlates with more severe disease, it does not seem useful as a marker of poorer response to treatment.

Our study has limitations. Firstly, the proportion of SAD in CTD-ILD is generated from patients diagnosed in the largest medical center in southwest of China, and any extrapolations of our findings to different populations need to be very careful. Secondly, the criteria used to evaluate SAD by PFTs varied in different studies [43, 44]. To minimize the interference by ethnic background, we chose the definition of SAD and reference values commonly used in the Chinese population studies [8].Thirdly, only PFTs data from CTD-ILD patients in this real-world study were available to evaluate SAD. Our findings need to be verified by other SAD assessment methods, including end-expiratory high resolution computed tomography (HRCT), impulse oscillometry (IOS) and lung clearance index (LCI) using nitrogen washout, pathological analysis etc., are required to confirm our study findings [45], [46]. Fourthly, previous studies showed differences in terms of estimated prevalence of ILD, respiratory symptoms and manifestations among different subtypes of CTD-ILD [3]. Our study on SAD was carried out from a mixed CTD-ILD population, although specific subtypes of CTD-ILD analysis were performed, the results are needed be cautious as the small number of each subtypes of CTD-ILD. Fifthly, our cohort study has a relatively high loss to follow-up rate which is hardly to be avoid due to the COVID-19 pandemic.

In conclusion, we carried out the assessment of SAD by PFTs in CTD-ILD and provide first evidence that SAD is a frequent finding in these patients. The presence of SAD could be used as a complementary parameter to assess disease severity in CTD-ILD patients, although it does not seem to affect treatment response. Our findings highlight the need for further studies on SAD in CTD-ILD, to validate our PFTs fundings by other methods, and to explore more in depth its pathobiological mechanisms and clarify its prognostic significance.

## Supplementary Information

**Additional file 1:** **Table S1.** The difference of prediction equations and reference values of different races. **Table S2.** Demographic and clinical characteristics of the retrospective study population. **Table S3.** Logistic regression for assessment of the factors associated with CTD-ILD with SAD. **Table S4.** Comparison of demographic and clinical characteristics between CTD-ILD patients with and without small airway dysfunction for prospective data. **Table S5.** Correlations between the PFT values and pulmonary symptoms. **Table S6.** Comparison of treatment between CTD-ILD patients with and without small airway dysfunction for prospective data (87 patients who finished two follow-up visits). **Table S7.** Changes of pulmonary function parameters versus baseline in CTD-ILD with and without SAD. **Table S8.** Interaction between SAD and trends of pulmonary function among different CTD-ILDs.

## Data Availability

The datasets used and/or analyzed during the current study are available from the corresponding author on reasonable request.

## Abbreviations

CTD-ILD: Connective tissue disease-related interstitial lung disease
DLCO: Diffusion capacity for carbon monoxide
FEF: Forced expiratory velocity
ILD-GAP Index: Interstitial lung disease–gender-age-physiology index
IIM: Idiopathic inflammatory myopathies
MMEF: Maximum mid expiratory flow
PFTs: Pulmonary function tests
RA: Rheumatoid arthritis
SAD: Small airway dysfunction
SSc: Systemic sclerosis

## References

[1] Vacchi C, Sebastiani M, Cassone G, Cerri S, Della Casa G, Salvarani C (2020). Therapeutic options for the treatment of interstitial lung disease related to connective tissue diseases. a narrative review. J Clin Med.

[2] Jee AS, Corte TJ (2019). Current and emerging drug therapies for connective tissue disease-interstitial lung disease (CTD-ILD). Drugs.

[3] Jee AS, Sheehy R, Hopkins P, Corte TJ, Grainge C, Troy LK (2021). Diagnosis and management of connective tissue disease-associated interstitial lung disease in Australia and New Zealand: A position statement from the Thoracic Society of Australia and New Zealand. Respirology (Carlton, Vic).

[4] Koo SM, Kim SY, Choi SM, Lee HK (2019). Korean guidelines for diagnosis and management of interstitial lung diseases: Part 5. connective tissue disease associated interstitial lung disease. Tuberculosis Respir Diseases.

[5] Kondoh Y, Makino S, Ogura T, Suda T, Tomioka H, Amano H (2021). 2020 guide for the diagnosis and treatment of interstitial lung disease associated with connective tissue disease. Respir Investig.

[6] Stanojevic S, Kaminsky DA, Miller M, Thompson B, Aliverti A, Barjaktarevic I, et al. ERS/ATS technical standard on interpretive strategies for routine lung function tests. Eur Respir J. 2022;60(1):2101499. 10.1183/13993003.01499-2021 34949706

[7] Dempsey TM, Scanlon PD (2018). Pulmonary Function Tests for the Generalist: A Brief Review. Mayo Clin Proc.

[8] Xiao D, Chen Z, Wu S, Huang K, Xu J, Yang L (2020). Prevalence and risk factors of small airway dysfunction, and association with smoking, in China: findings from a national cross-sectional study. Lancet Respir Med.

[9] van den Bosch WB, James AL, Tiddens H. Structure and function of small airways in asthma patients revisited. Eur Respir Rev: Off J Eur Respir Soc. 2021;30(159):200186. 10.1183/16000617.0186-2020 PMC9488985 33472958

[10] Spagnolo P, Ryerson CJ, Putman R, Oldham J, Salisbury M, Sverzellati N (2021). Early diagnosis of fibrotic interstitial lung disease: challenges and opportunities. Lancet Respir Med.

[11] Cazzola M, Calzetta L, Matera MG (2021). Long-acting muscarinic antagonists and small airways in asthma: Which link?. Allergy.

[12] Verleden SE, Tanabe N, McDonough JE, Vasilescu DM, Xu F, Wuyts WA (2020). Small airways pathology in idiopathic pulmonary fibrosis: a retrospective cohort study. Lancet Respir Med.

[13] Ikezoe K, Hackett TL, Peterson S, Prins D, Hague CJ, Murphy D (2021). Small airway reduction and fibrosis is an early pathologic feature of idiopathic pulmonary fibrosis. Am J Respir Crit Care Med.

[14] Evans CM, Fingerlin TE, Schwarz MI, Lynch D, Kurche J, Warg L (2016). Idiopathic pulmonary fibrosis: a genetic disease that involves mucociliary dysfunction of the peripheral airways. Physiol Rev.

[15] van den Hoogen F, Khanna D, Fransen J, Johnson SR, Baron M, Tyndall A (2013). 2013 classification criteria for systemic sclerosis: an American college of rheumatology/European league against rheumatism collaborative initiative. Arthritis Rheum.

[16] Ciang NC, Pereira N, Isenberg DA (2017). Mixed connective tissue disease-enigma variations?. Rheumatology (Oxford).

[17] Bohan A, Peter JB (1975). Polymyositis and dermatomyositis (first of two parts). N Engl J Med.

[18] Wilfong EM, Lentz RJ, Guttentag A, Tolle JJ, Johnson JE, Kropski JA (2018). Interstitial pneumonia with autoimmune features: an emerging challenge at the intersection of rheumatology and pulmonology. Arthritis Rheumatol (Hoboken, NJ).

[19] Arnett FC, Edworthy SM, Bloch DA, McShane DJ, Fries JF, Cooper NS (1988). The American rheumatism association 1987 revised criteria for the classification of rheumatoid arthritis. Arthritis Rheum.

[20] Hochberg MC (1997). Updating the American college of rheumatology revised criteria for the classification of systemic lupus erythematosus. Arthritis Rheum.

[21] Ryerson CJ, Vittinghoff E, Ley B, Lee JS, Mooney JJ, Jones KD (2014). Predicting survival across chronic interstitial lung disease: the ILD-GAP model. Chest.

[22] Graham BL, Steenbruggen I, Miller MR, Barjaktarevic IZ, Cooper BG, Hall GL (2019). Standardization of Spirometry 2019 Update. An Official American Thoracic Society and European Respiratory Society Technical Statement. American J Respir Critical Care Med.

[23] Pellegrino R, Viegi G, Brusasco V, Crapo RO, Burgos F, Casaburi R (2005). Interpretative strategies for lung function tests. Eur Respir J.

[24] Jian W, Gao Y, Hao C, Wang N, Ai T, Liu C (2017). Reference values for spirometry in Chinese aged 4–80 years. J Thorac Dis.

[25] Jr McFadden ER, Linden DA (1972). A reduction in maximum mid-expiratory flow rate A spirographic manifestation of small airway disease. American J Med.

[26] Postma DS, Brightling C, Baldi S, Van den Berge M, Fabbri LM, Gagnatelli A (2019). Exploring the relevance and extent of small airways dysfunction in asthma (ATLANTIS): baseline data from a prospective cohort study. Lancet Respir Med.

[27] Usmani OS, Singh D, Spinola M, Bizzi A, Barnes PJ (2016). The prevalence of small airways disease in adult asthma: A systematic literature review. Respir Med.

[28] Crisafulli E, Pisi R, Aiello M, Vigna M, Tzani P, Torres A (2017). Prevalence of small-airway dysfunction among copd patients with different gold stages and its role in the impact of disease. Respir; Intern Review Thoracic Diseases.

[29] Bonini M, Usmani OS (2015). The role of the small airways in the pathophysiology of asthma and chronic obstructive pulmonary disease. Ther Adv Respir Dis.

[30] Basil MC, Cardenas-Diaz FL, Kathiriya JJ, Morley MP, Carl J, Brumwell AN (2022). Human distal airways contain a multipotent secretory cell that can regenerate alveoli. Nature.

[31] Zhang X, Xie B, Ban C, Ren Y, Ye Q, Zhu M (2022). Small airway dysfunction in Chinese patients with idiopathic pulmonary fibrosis. BMC Pulm Med.

[32] Burgel PR, Bergeron A, de Blic J, Bonniaud P, Bourdin A, Chanez P (2013). Small airways diseases, excluding asthma and COPD: an overview. European Respir Rev : an official journal of the European Respiratory Society.

[33] Shao T, Shi X, Yang S, Zhang W, Li X, Shu J (2021). Interstitial Lung Disease in Connective Tissue Disease: A Common Lesion With Heterogeneous Mechanisms and Treatment Considerations. Front Immunol.

[34] Gupta S, Ferrada MA, Hasni SA (2019). Pulmonary manifestations of primary sjögren's syndrome: underlying immunological mechanisms, clinical presentation, and management. Front Immunol.

[35] García-Quero C, García-Río F (2021). Smoking-Induced Small Airway Dysfunction. An Early Marker of Future Copd?. Arch Bronconeumol.

[36] van der Lee I, Zanen P, Biesma DH, van den Bosch JM (2005). The effect of red cell transfusion on nitric oxide diffusing capacity. Respiration; international review of thoracic diseases.

[37] van Westerloo DJ, Giebelen IA, Florquin S, Bruno MJ, Larosa GJ, Ulloa L (2006). The vagus nerve and nicotinic receptors modulate experimental pancreatitis severity in mice. Gastroenterology.

[38] Qiu F, Liang CL, Liu H, Zeng YQ, Hou S, Huang S (2017). Impacts of cigarette smoking on immune responsiveness: Up and down or upside down?. Oncotarget.

[39] Sun Z, Smyth K, Garcia K, Mattson E, Li L, Xiao Z (2013). Nicotine inhibits memory CTL programming. PLoS ONE.

[40] Gao M, Aveyard P, Lindson N, Hartmann-Boyce J, Watkinson P, Young D (2022). Association between smoking, e-cigarette use and severe COVID-19: a cohort study. Int J Epidemiol.

[41] Oldham JM, Lee CT, Wu Z, Bowman WS, Vu Pugashetti J, Dao N, et al. Lung function trajectory in progressive fibrosing interstitial lung disease. Eur Respir J. 2022;59(6):2101396. 10.1183/13993003.01396-2021 PMC10039317 34737223

[42] Goto Y, Tobino K, Okahisa M, editors. Significance of small airway obstruction in patients with idiopathic pulmonary fibrosis. ERS International Congress. 2019. PA1336.

[43] Morris ZQ (2014). In Reply: Isolated Reduction of the FEV(3)/FVC Ratio as an Indicator of Mild Airflow Obstruction. Chest.

[44] Mori S, Koga Y, Sugimoto M (2011). Small airway obstruction in patients with rheumatoid arthritis. Mod Rheumatol.

[45] McNulty W, Usmani OS (2014). Techniques of assessing small airways dysfunction. European Clin Respir J.

[46] Hansell DM, Bankier AA, MacMahon H, McLoud TC, Müller NL, Remy J (2008). Fleischner Society: glossary of terms for thoracic imaging. Radiology.

