# Supplementary material for: Monitoring small airway dysfunction in connective tissue disease-related interstitial lung disease: a retrospective and prospective study
Source: BMC Pulm Med. 2023 Mar 20;23:90. doi: 10.1186/s12890-023-02381-z (PMC10026226; doi:10.1186/s12890-023-02381-z)
Supplement: Supplementary file 1 — Additional file 1: Table S1. The difference of prediction equations and reference values of different races. Table S2. Demographic and clinical characteristics of the retrospective study population. Table S3. Logistic regression for assessment of the factors associated with CTD-ILD with SAD. Table S4. Comparison of demographic and clinical characteristics between CTD-ILD patients with and without small airway dysfunction for prospective data. Table S5. Correlations between the PFT values and pulmonary symptoms. Table S6. Comparison of treatment between CTD-ILD patients with and without small airway dysfunction for prospective data (87 patients who finished two follow-up visits). Table S7. Changes of pulmonary function parameters versus baseline in CTD-ILD with and without SAD. Table S8. Interaction between SAD and trends of pulmonary function among different CTD-ILDs. [file 12890_2023_2381_MOESM1_ESM.docx]

**Supplementary materials**

Table S1 The difference of prediction equations and reference values of different races.

|  | China^1^ | GLI equation for East Asia^2^ |
| --- | --- | --- |
| Population | 7115 (4 to 80 yrs) | 4992 Northeast Asian, 8255 Southeast Asia (3 to 95 yrs) |
| Regions involving China | Northeast, North, Northwest, Southwest, South, East and Central China | Northeast Asia (NEA): northeast China  Southeast Asia (SEA): Hong Kong, Taiwan, Shenzhen |
| Advantages | Covering all main regions of China;  More representative and better interpretation for Chinese |  |
| Disadvantages |  | Only involved several regions in China;  Devided into 2 equations (NEA-GLI and SEA-GLI), not convenient for the unity and comparison in the clinical application;  2 studies with large population in China indicates the underestimation of SEA-GLI equation;  After validated with Chinese measured spirometry data, popular Caucasian reference values adjusted with ethnic conversion factors were inappropriate for Chinese. |

1. Jian W, Gao Y, Hao C, Wang N, Ai T, Liu C, Xu Y, Kang J, Yang L, Shen H, et al: **Reference values for spirometry in Chinese aged 4-80 years.** *J Thorac Dis* 2017, **9:**4538-4549.

2. Quanjer PH, Stanojevic S, Cole TJ, Baur X, Hall GL, Culver BH, Enright PL, Hankinson JL, Ip MS, Zheng J, Stocks J: **Multi-ethnic reference values for spirometry for the 3-95-yr age range: the global lung function 2012 equations.** *Eur Respir J* 2012, **40:**1324-1343.

Table S2. Demographic and clinical characteristics of the retrospective study population.

|  | Retrospective study  (N = 491) |
| --- | --- |
| Age, (y) | 54.41±13.02 |
| Female, n (%) | 338 (68.8%) |
| BMI, (kg/m2) | 22.58±3.51 |
| Ever smoker, n (%) | 113 (23.0%） |
| SAD, n (%) | 233 (47.5%) |
| Type of CTD |  |
| RA | 63 (12.8%) |
| IIM | 116 (23.6%) |
| pSS | 55 (11.2%) |
| SSc | 96 (19.6%) |
| SLE | 18 (3.7%) |
| OS-ILD | 63 (12.8%) |
| other types | 80 (16.3%) |
| Patterns of CTD-ILD |  |
| NSIP | 231 (47.1%) |
| UIP | 73 (14.9%) |
| OP | 9 (1.8%) |
| LIP | 4 (0.8%) |
| Others | 174 (35.4%) |
| ILD-GAP index |  |
| 0-1 | 405 (82.5%) |
| 2-3 | 78 (15.9%) |
| 4-5 | 8 (1.6%) |
| ＞5 | 0 (0%) |
| Pulmonary function tests |  |
| FVC, (%Pred) | 75.43±20.31 |
| FEV1/FVC, (%) | 82.55±11.72 |
| FEV1, (%Pred) | 77.96±21.11 |
| DLCO, (%Pred) | 61.44±21.10 |
| MMEF, (%Pred) | 67.56±26.95 |
| FEF50, (%Pred) | 73.49±28.55 |
| FEF75, (%Pred) | 61.36±34.03 |

Data are presented as n (%) or means ± SD. SD, standard deviation; BMI, Body mass index; SAD, small airway disease; RA, arthritis; IIM, idiopathic inflammatory myopathies; pSS, primary Sjogren’s syndrome; SSc, systemic sclerosis; SLE, lupus erythematosus; OS, overlap syndrome; ILD-GAP, interstitial lung disease–gender-age-physiology; FEV1: forced expiratory volume in the first second; FVC: forced vital capacity; DLCO diffusion capacity for carbon monoxide; MMEF, maximum mid expiratory flow; FEF, forced expiratory velocity.

Table S3. Logistic regression for assessment of the factors associated with CTD-ILD with SAD.

|  | Univariate analysis | | Multivariate analysis | |
| --- | --- | --- | --- | --- |
| Variables | OR (95% CI) | P | OR (95% CI) | P |
| Gender (Male vs Female) | 1.625 (1.102-2.396) | 0.014 | 2.170（1.204-3.914） | 0.010 |
| Smoking (No vs Yes) | 0.552 (0.358-0.851) | 0.007 | - | 0.601 |
| Age, (y) | 1.011 (0.998-1.025) | 0.103 | 1.025 (1.005-1.046) | 0.017 |
| BMI | 0.941 (0.875-1.011) | 0.096 | - | 0.055 |
| ILD duration, (y) | 0.958 (0.884-1.038) | 0.294 | - | - |
| CTD duration, (y) | 0.996 (0.969-1.024) | 0.785 | - | - |

OR, odds ratio; CI, confidence interval; BMI, body mass index.

Table S4. Comparison of demographic and clinical characteristics between CTD-ILD patients with and without small airway dysfunction for prospective data

|  | CTD-ILD with SAD（n = 60） | CTD-ILD without SAD（n = 79） | **P** |
| --- | --- | --- | --- |
| Age, (y) | 51.85±10.74 | 53.30±11.75 | 0.455 |
| Female, n (%) | 56（93.3%） | 60（75.9%） | 0.006 |
| BMI, (kg/m2) | 22.97±3.25 | 22.54±2.98 | 0.420 |
| Ever smoker, n (%) | 4（6.7%） | 18（22.8%） | 0.010 |
| Times of acute exacerbation in the last year | 1.07±1.163 | 0.80±1.114 | 0.168 |
| **Pulmonary function tests** |  |  |  |
| FVC, (%Pred) | 76.16±22.03 | \| 87.94±20.67 \| 87.94 \| \| --- \| --- \| | 0.003 |
| FEV1/FVC, (%) | 81.87±5.81 | 88.16±6.45 | <0.001 |
| FEV1, (%Pred) | 74.42±17.49 | 91.89±20.67 | <0.001 |
| DLCO, (%Pred) | 64.21±18.96 | 69.39±18.02 | 0.105 |

Data are presented as n (%) or means ± SD. SD, standard deviation; BMI, Body mass index.

FEV1: forced expiratory volume in the first second; FVC: forced vital capacity; DLCO diffusion capacity for carbon monoxide.

Table S5. Correlations between the PFT values and pulmonary symptoms.

|  | **mMRC** | **Borg** | **VAS** | **LCQ** |
| --- | --- | --- | --- | --- |
| **FVC%**  rho  p-value | -0.364  0.001 | -0.344  0.001 | -0.296  0.005 | 0.303  0.004 |
| **DLCO%**  rho  P-value | -0.368  <0.001 | -0.380  <0.001 | -0.208  0.054 | 0.241  0.025 |

FVC: forced vital capacity; DLCO diffusion capacity for carbon monoxide; MMEF, maximum mid-expiratory flow; mMRC, modified Medical Research Council for dyspnea; VAS, Visual Analog Scale; LCQ, Leicester Cough Questionnaire for cough.

Table S6. Comparison of treatment between CTD-ILD patients with and without small airway dysfunction for prospective data (87 patients who finished two follow-up visits)

|  | CTD-ILD with SAD（n = 36） | CTD-ILD without SAD（n = 51） | **P** |
| --- | --- | --- | --- |
| **Immunosuppressive therapies, n (%)** |  |  |  |
| None | 1（2.8%） | 3（5.9%） | 0.793 |
| Corticosteroids alone | 2（5.6%） | 2（3.9%） |  |
| One immunosuppressive alone | 0（0.0%） | 2（3.9%） |  |
| Two immunosuppressive | 1（2.8%） | 0（0.0%） |  |
| Corticosteroids combined with one immunosuppressive | 18（50.0%） | 23（45.1%） |  |
| Corticosteroids combined with two immunosuppressive | 13（36.1%） | 19（37.3%） |  |
| Corticosteroids combined with three immunosuppressive | 1（2.8%） | 2（3.9%） |  |
| **Antifibrotic therapy, n (%)** | 27（75.0%） | 30（58.8%） | 0.169 |
| **Acetylcysteine, n (%)** | 21（58.3%） | 29（56.9%） | 0.891 |

Immunosuppressive included methotrexate, azathioprine, cyclophosphamide, tacrolimus, mycophenolate mofetil, hydroxychloroquine , leflunomide, tofanib, and cyclosporin. Antifibrotic therapy included nintedanib and pirfenidone.

Table S7. Changes of pulmonary function parameters versus baseline in CTD-ILD with and without SAD.

|  | **Changes at 6 month** | | **Changes at 12 month** | |
| --- | --- | --- | --- | --- |
|  | SAD  (n=36) | No SAD  (n=51) | SAD  (n=36) | No SAD  (n=51) |
| **FVC%** | 2.16 ± 0.98  (-0.293-4.615), p=0.100 | 3.62 ± 1.36 (0.258-6.989) p=0.013 | 6.37 ± 1.95 (1.473-11.266), p=0.007 | 5.13 ± 1.84 (0.561-9.694), p=0.023 |
| **DLCO%** | -0.68 ± 1.15  (-3.579-2.219),  p=1.000 | -0.78 ± 1.14  (-3.603-2.039),  p=1.000 | -1.54 ± 1.84  (-6.181-3.094),  p=1.000 | -0.31 ± 2.31  (-6.019-5.399),  p=1.000 |
| **MMEF%** | 2.05 ± 1.66  (-2.121-6.229),  p=0.671 | -0.79 ± 2.42  (-6.784-5.212),  p=1.000 | 1.53 ± 3.17  (-6.448-9.517),  p=1.000 | -0.85 ± 2.89  (-8.028-6.326),  p=1.000 |
| **FEF75%** | 0.96 ± 1.53  (-2.887-4.807),  p=1.000 | -2.01 ± 2.64  (-8.549-4.528),  p=1.000 | 1.62 ± 2.56  (-4.834-8.074),  p=1.000 | 1.57 ± 3.28  (-6.571-9.710),  p=1.000 |
| **FEF50%** | 2.30 ± 1.85  (-2.342-6.948),  p=0.661 | 0.05 ± 2.71  (-6.670-6.780),  p=1.000 | 3.83 ± 2.63  (-2.792-10.449),  p=0.463 | 0.61 ± 3.09  (-7.045-8.265),  p=1.000 |

Data are presented as n (%) or means ± SD (95% CI). SD, standard deviation; CI, confidence interval; SAD: small airway dysfunction; FVC: forced vital capacity; DLCO diffusion capacity for carbon monoxide; MMEF, maximum mid-expiratory flow; FEF, forced expiratory flow.

Table S8. Interaction between SAD and trends of pulmonary function among different CTD-ILDs.

|  |  | F | P |
| --- | --- | --- | --- |
| **FVC%** | IIM-ILD  (N = 28) | 0.984 | 0.357 |
|  | pSS-ILD  (N = 6) | 0.054 | 0.850 |
|  | SSc-ILD  (N = 17) | 0.944 | 0.360 |
|  | OS-ILD  (N = 9) | 0.316 | 0.615 |
| **DLCO%** | IIM-ILD  (N = 28) | 0.712 | 0.457 |
|  | pSS-ILD  (N = 6) | 1.998 | 0.229 |
|  | SSc-ILD  (N = 17) | 2.263 | 0.151 |
|  | OS-ILD  (N = 9) | 0.225 | 0.713 |

SAD, small airway disease; IIM, idiopathic inflammatory myopathies; pSS, primary Sjogren’s syndrome; SSc, systemic sclerosis; OS, overlap syndrome; FVC: forced vital capacity; DLCO diffusion capacity for carbon monoxide.
